# Hyaluronic Acid Modified Metal Nanoparticles and Their Derived Substituents for Cancer Therapy: A Review

**DOI:** 10.3390/pharmaceutics15061713

**Published:** 2023-06-12

**Authors:** Uluvangada Thammaiah Uthappa, Maduru Suneetha, Kanalli V. Ajeya, Seong Min Ji

**Affiliations:** 1 School of Chemical Engineering, Yeungnam University, 280 Daehak-Ro, Gyeongsan 38541, Republic of Korea; msunithachem@gmail.com; 2 Department of Bioengineering, Saveetha School of Engineering, Saveetha Institute of Medical and Technical Sciences, Chennai 602105, India; 3 Department of Environment and Energy Engineering, Chonnam National University, 77 Yongbong-Ro, Buk-gu, Gwangju 61186, Republic of Korea; ajeyhegde94@gmail.com

**Keywords:** biopolymers, metal nanoparticles, targeted therapy, controlled release, biomaterials, hyaluronic acid

## Abstract

The use of metal nanoparticles (M-NPs) in cancer therapy has gained significant consideration owing to their exceptional physical and chemical features. However, due to the limitations, such as specificity and toxicity towards healthy cells, their application in clinical translations has been restricted. Hyaluronic acid (HA), a biocompatible and biodegradable polysaccharide, has been extensively used as a targeting moiety, due to its ability to selectively bind to the CD44 receptors overexpressed on cancer cells. The HA-modified M-NPs have demonstrated promising results in improving specificity and efficacy in cancer therapy. This review discusses the significance of nanotechnology, the state of cancers, and the functions of HA-modified M-NPs, and other substituents in cancer therapy applications. Additionally, the role of various types of selected noble and non-noble M-NPs used in cancer therapy are described, along with the mechanisms involved in cancer targeting. Additionally, the purpose of HA, its sources and production processes, as well as its chemical and biological properties are described. In-depth explanations are provided about the contemporary applications of HA-modified noble and non-noble M-NPs and other substituents in cancer therapy. Furthermore, potential obstacles in optimizing HA-modified M-NPs, in terms of clinical translations, are discussed, followed by a conclusion and future prospects.

## 1. Introduction

The rapid expansion of nanotechnology is attributed to multidisciplinary collaboration among researchers from academic, industrial, and federal sectors [1,2]. Nanotechnology is a rapidly developing field that has the potential to impact many facets of our lives, including medicine. This field includes nanoparticles (NPs), which are substances with at least one dimension smaller than 100 nm [3], categorized by exceptional physicochemical, functional and biological features [4,5]. In the pharmaceutical field, NPs are utilized to enhance the biodistribution of drugs, or to target them to particular cells or locations. The applications of these systems are extensively used in several biomedical applications, such as tissue engineering, hyperthermia, biosensors, and laboratory diagnostics etc. [1].

As the top cause of illness and mortality worldwide, cancer diseases are recognized as fatal malignancies. In 2020 alone, cancer resulted in approximately 9.9 million deaths and 19.2 million new cases worldwide. It is estimated that by 2040, the global cancer burden will rise to between 29–37 million new cases [6]. The development of cancer is linked to abnormalities in the genes that regulate the balance between cell proliferation and cell death necessary for cellular homeostasis. When these regulatory genes are defective, an imbalance in the cell cycle and apoptosis occurs, leading to uncontrolled cell growth, dysfunction of cellular tissue, invasion of neighboring cells by tumors, and, eventually, the progression of the disease to metastasis. Cancer cells are known for their aggressive cell proliferation and ability to evade apoptosis, setting them apart from non-cancerous cells [7].

Due to the drastic and unfavorable properties and substandard treatment results associated with conventional therapeutic methods, such as chemotherapy, radiotherapy, and surgery, there has been a significant shift in research towards the integration of nanotechnological approaches in cancer management [8,9,10,11]. In the past ten years, there has been a substantial surge of interest in the utilization of nanotechnology as an interdisciplinary strategy for cancer theranostics, resulting in an exponential growth in the number of researchers dedicated to the development of tumor-targeting NPs [12,13]. Among various NPs, the M-NPs have received significant attention due to their potential to serve as versatile agents. They are particularly prominent in current cancer research platforms which have gained significant attention. Numerous studies have indicated that M-NPs can be utilized to treat cancers, with preliminary results and clinical trials which are currently progressing. The use of non-noble metal-based cancer therapy has the potential to progress towards more cost-effective treatments when compared to expensive chemotherapeutic approaches [14]. Such properties of M-NPs make them particularly attractive in the field of cancer therapy. These properties include a comparatively narrow size and shape distribution, a long activity period, surface functionalization, and the ability to utilize optical or heat-based therapeutic approaches [15,16]. M-NPs have demonstrated enhanced targeting, gene silencing, and drug delivery capabilities. In particular, functionalizing the M-NPs with targeting ligands can promote precise deposition into tumor cells, specifically benefitting various cancer treatments [17]. On the other hand, there has been a rapidly growing interest in natural polymers (ex: hyaluronic acid (HA)) due to their inherent biocompatibility, biodegradability, targeting capability and non-immunogenicity. These characteristics are crucial for the development of effective cancer therapeutic systems. Thus, by surface modifications of the HA with M-NPs multifunctional systems can be developed in order to achieve superior therapeutic efficacies in cancer therapy [18,19,20].

To our knowledge, there are some reviews published individually on HA, M-NPs, HA and other NPs for cancer therapy and biomedical applications [21,22,23,24,25,26]. However, to date, there are no reviews available on HA-modified selected noble and non-noble M-NPs and other substituents for cancer therapy. In this review, with the importance of nanotechnology, an overview of the current status of cancers and the role of HA-modified M-NPs and other substituents are discussed. The core part of this review article is to educate researchers, and, specifically, those conducting research on nanotechnology-assisted cancer therapy, as to the significance of HA-modified M-NPs and their other substituents in cancer therapy. The mechanisms involved in cancer targeting, different types of selected noble and non-noble M-NPs used in cancer therapy are also addressed. In addition, the role of HA, its sources and fabrication methods, and its chemical and biological properties are described. Furthermore, aspects of past and recent applications of HA-modified noble and non-noble M-NPs and other substituents in cancer therapy are thoroughly elucidated. Lastly, potential challenges involved in HA-modified M-NPs for further optimization, with regard to clinical translations, are discussed, followed by a conclusion and future prospects.

## 2. Noble M-NPs Used for Cancer Therapy

The utilization of different types of M-NPs in cancer treatment has been widely explored. These M-NPs can be generally classified into two categories based on their chemical properties: noble and non-noble M-NPs. In the below section, a brief outline of noble M-NPs is provided.

### 2.1. Gold (Au-NPs)

Au is recognized as a noble element because of its non-reactive characteristics, which enable it to withstand chemical oxidation, degradation, and corrosion, preserving its nature for prolonged periods, even for thousands of years. Au-NPs can be produced through different methods, including chemical, physical, biological, and green synthesis or even utilizing both bottom-up and top-down approaches. The exclusive physicochemical features of Au-NPs make them appropriate in cancer applications [27].

### 2.2. Silver (Ag-NPs)

The main modes of action by which Ag-NPs exert their effects include inducing oxidative stress, causing DNA rupture, and generating reactive oxygen species (ROS). ROS play a critical role in maintaining cellular homeostasis by regulating various signaling pathways. These highly reactive molecules are produced as byproducts of cellular metabolism. Nonetheless, when present in excess, intracellular ROS can trigger oxidative stress and induce impairment to cellular components, such as DNA, lipids, and proteins, thereby contributing to Ag-NP-induced toxicity [28]. Ag-NPs are known to induce toxicity in treated cells by releasing silver ions into the cytosol after endocytosis and subsequent breakdown of the nanoparticles in acidic environments. Consequently, Ag-NPs have been associated with an elevated risk of cancer and cell death, owing to their capacities to disrupt vital metabolic and cell cycle pathways in cells [29].

### 2.3. Platinum (Pt-NPs)

Pt-based drugs, such as cisplatin, carboplatin, and oxaliplatin, are commonly used in cancer treatment for patients worldwide. Nevertheless, due to the drugs’ lack of specificity towards cancer cells, they can cause adverse reactions and contribute to the progress of drug resistance [30]. Coating the surface of Pt-NPs with a biocompatible substance (such as HA) could potentially enhance the therapeutic efficacy by prolonging the circulation time in the body [31].

### 2.4. Palladium (Pd-NPs)

Researchers have highlighted the exceptional catalytic and optical properties of Pd-NPs, making them suitable for theragnostic applications. Pd-NPs have been utilized as prodrug activators, and photothermal agents, as well as anticancer agents [14].

## 3. Non-Noble M-NPs Used for Cancer Therapy

Despite being prone to oxidation, non-noble metals offer several advantages, due to their low cost, abundance, localized therapy, enhanced side effects, and excellent conductivity. The following section describes the characteristic features of selected non-noble M-NPs used in cancer therapy.

### 3.1. Magnetic NPs

Manipulating magnetic NPs is made possible by the application of external magnetic fields [32]. Owing to their exceptional features, such as ease of synthesis, low toxicity and good biodegradability, they have gained significant attention in cancer therapy [33].

### 3.2. Zinc Oxide (Zn-NPs)

Zn-NPs are among the most frequently occurring metallic NPs found globally, and their ability to generate ROS when exposed to light has recently gained significant attention. Chemical modification with biopolymers can enhance their photocatalytic effectiveness and ROS generation capacities [34].

### 3.3. Cerium Oxide (Ce-NPs)

Due to the peculiar chemistry of cerium oxide, cerium oxide NPs (Ce-NPs) have been considered as potential anti-cancer agents [35]. It has been suggested that Ce-NPs have enormous potential value in cancer treatment. They exhibit synergistic cytotoxicity when combined with chemotherapeutics because their mode of action is thought to be through the production of intracellular ROS. The use of Ce-NPs raises serious safety concerns, due to a propensity to aggregate and cause unfavorable side effects. Thus, to address such demerits, Ce-NPs can be conjugated with HA to decrease agglomeration and to improve their biological activities [36,37].

## 4. Mechanism of Cancer Targeting

It is worth noting that M-NPs have been found to exhibit antitumor activity. The below paragraph outlines the typical mechanisms of action utilized in cancer treatments.

### Active or Passive Targeting of Tumor

The use of M-NPs in cancer therapy can improve the concentration of therapeutic agents through passive and active mechanisms. In particular, tumor vasculatures often display irregular branching and leaky areas, with pore sizes ranging from 100 nm to several hundred nanometers, attributed to the decreased presence of pericytes resulting from the swift proliferation of endothelial cells. This phenomenon is commonly observed in cancer treatments [38]. The increased permeability and retention (EPR) effect refers to the phenomenon of the body concentrating inert M-NPs in the tumor due to its leaky vasculature, which is a result of the passive targeting mechanism. Alternatively, active targeting modifies the surface of M-NPs to functionally enhance the therapeutic delivery system, resulting in selective tissue targeting [39]. Incorporating tumor-targeting ligands into M-NPs could lead to the release of drugs targeted specifically to the tumor site [40]. Other approaches or mechanisms involved in cancer therapy, such as tumor targeting through gene silencing, drug delivery through NPs, NP-based hyperthermia and radiotherapy treatment using NPs are thoroughly explained in a recent review article published by Xu et al. For more detailed information readers may refer to this article [14].

## 5. Hyaluronic Acid (HA) in Cancer Therapy

### 5.1. Sources and Preparations

**Figure 1:**
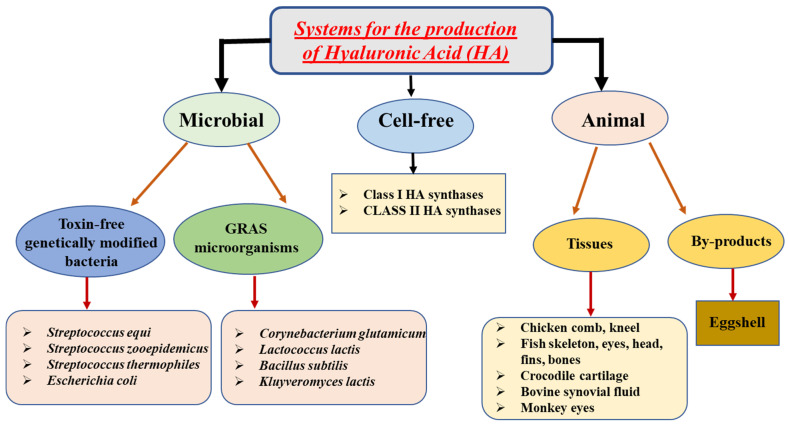
Various approaches/sources of HA. Reproduced with modifications from [43].

HA can be derived from various sources, including microorganisms, cell-free systems, and animal tissues, as illustrated in Figure 1. Among these, animal tissues, such as skin, eyes, and synovial fluid, are commonly used to obtain HA. This approach involves several steps, such as enzymatic breakdown, removal of unwanted proteins, precipitation using alcohol and quaternary ammonium salt dehydration and separation. However, this method has low efficiency, and the scarcity and quality of raw materials pose significant challenges. Additionally, it is expensive, and the extracted HA can trigger immune responses and infectious diseases. Furthermore, the extraction process necessitates large amounts of toxic chemicals, such as acids and salt chemical reagents, which can pollute the environment and must be removed from the final product. Consequently, alternative methods of production, particularly microbial fermentation of HA, have gained popularity [41]. The bacterial-produced HA is similar in quality to animal-derived HA, but, unlike the latter, it does not trigger immune responses. Moreover, it is highly compatible with cells, making it an attractive option for biomedical applications. Several bacterial species, including strains that are genetically-modified and generally recognized as safe (GRAS), are used to manufacture HA. The S. zooepidemicus, which has low pathogenicity, is the primary strain employed for HA production through microbial fermentation [42].

**Figure 2:**
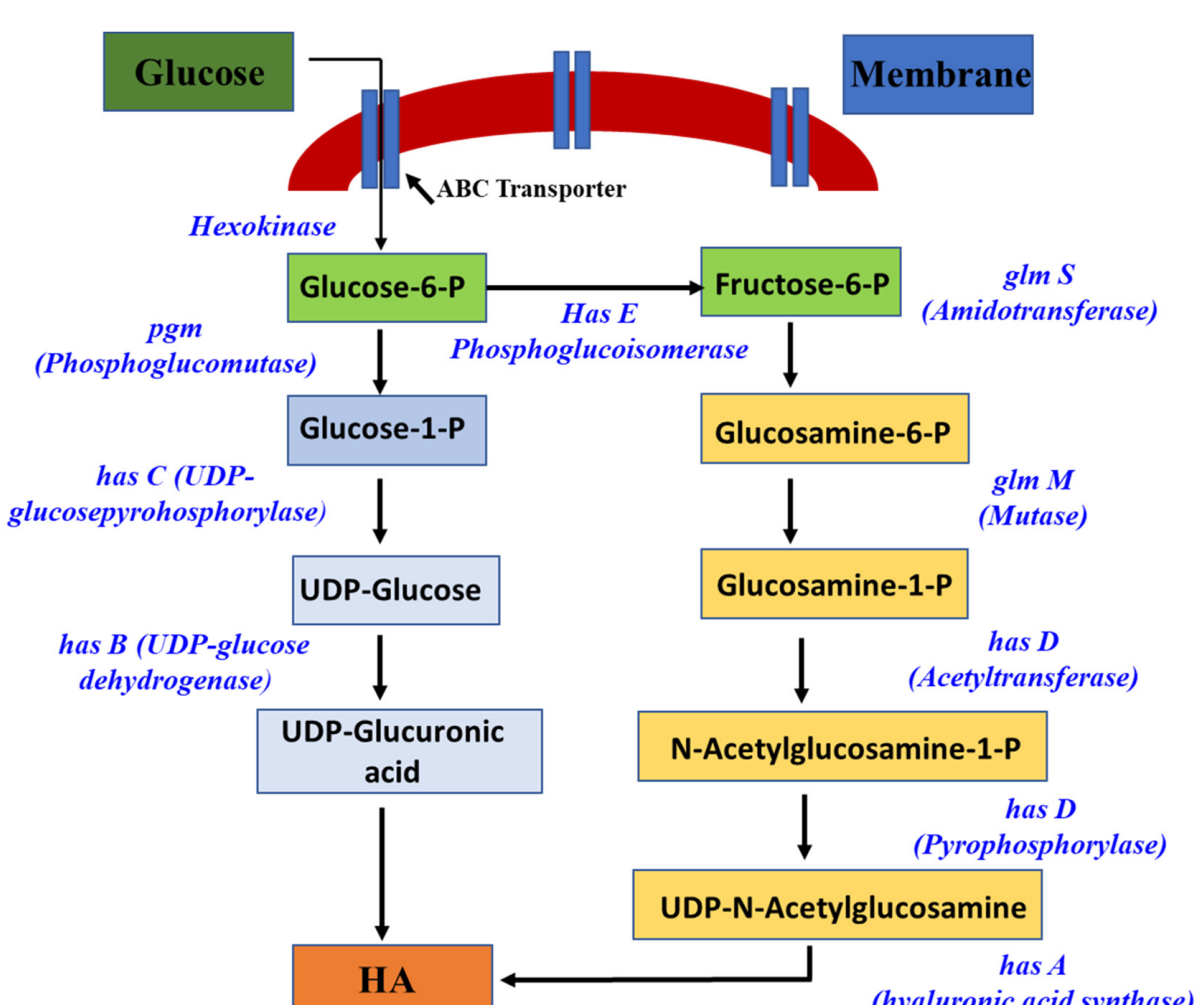
Schematic showing biosynthetic pathways. Reproduced with modifications from [43].

The biosynthesis of HA originates with the phosphorylation of glucose by hexokinase to generate glucose-6-phosphate. This initiates two distinct pathways for the synthesis [44]. The HA biosynthetic pathway is shown in Figure 2.

Cell-free systems represent an alternative approach for HA production; however, they remain suboptimal and unsuitable for large-scale industrial manufacturing. The Group A and C streptococci are known to possess Class I HAS enzymes that are characterized by their integral membrane structure containing 4–6 transmembrane domains and 1–2 membrane domains. These enzymes also possess the ability to be lipid-modified, which facilitates the extrusion of HA molecules outside the cell. Moreover, they can add UDP sugars from the reducing end of the HA chain to the developing HA polymer, making them a unique class of enzymes [45]. Various culture conditions, including pH, temperature, agitation speed, aeration rate, shear stress, dissolved oxygen, and bioreactor type, can have a significant impact on the regulation of HA production. Aerobic fermentation generally leads to higher HA concentrations and yields compared to anaerobic fermentation, due to several factors. One possible explanation for how oxygen affects HA synthesis is that it may stimulate the synthesis by protecting streptococcal cells from oxygen metabolites through the aggregation of cells mediated by their HA capsules. Another probable explanation is that oxygen may redirect carbon flux towards acetic acid, resulting in increased production of ATP [41,44]. Therefore, from some of these studies, it is evident that there is still a need for further improvements in the production of HA to facilitate its economic production for various applications requiring HA of different molecular weights. Using non-pathogenic and safe heterologous hosts, such as E. coli or B. subtilis, for bacterial fermentation could be a viable approach to producing HA. This method has the potential to generate HA molecules of different molecular weights. Furthermore, metabolic engineering strategies can be employed to improve and regulate the molecular weight of the produced HA [43].

### 5.2. Structure and Physical-Chemical Properties

#### 5.2.1. Chemistry Characteristics

HA is a biopolymer comprised of D-glucuronic acid and N-acetyl-D-glucosamine units [22,46]. HA, being hydrophilic, possesses hydroxyl groups, which enable it to form hydrogen bonds with water molecules. Additionally, the carboxyl, hydroxyl, and acetamido functional groups present on HA can be utilized for the purpose of chemical modifications [47]. HA is a biodegradable and biocompatible biopolymer which is used extensively in cancer therapy [48]. The chemical moieties of HA undergo deprotonation under physiological conditions as the carboxyl groups of HA have a pKa value of 3–4 [49]. The hydrophilic nature of HA allows it to form viscous and elastic gels through hydration, resulting in the binding of water molecules [50].

#### 5.2.2. Biological Characteristics

In more recent years, attention has been dedicated to natural polysaccharide polymers, owing to their various health promoting functions (such as improved pharmacological activity, and antioxidant, anticoagulant and anticancer properties) [51,52,53,54,55,56,57]. HA is considered a promising agent or natural polysaccharide polymer in cancer therapy, as it contains reactive sites, such as carboxylic, hydroxyl, and -NHCOCH_3_ groups, that can be covalently modified. Among these, the carboxylic groups are particularly useful for chemical modification through amination or esterification or conjugation with M-NPs and other substituents [58,59]. The binding affinity of HA to CD44 molecules expressed on cancer cells has made HA a promising tool in cancer therapy, and it has been widely used for this purpose [60]. The CD44 is a cell surface glycoprotein with multifunctional roles that include proliferation, migration, and angiogenesis. The binding ability of HA to CD44 allows it to internalize into cells, which makes it a promising candidate to suppress the progression of cancers [61].

## 6. Application of HA-Modified Noble M-NPs and Other Substituents in Various Cancer Therapies

### 6.1. HA-Modified Au-M-NPs and Other Substituents

**Figure 3:**
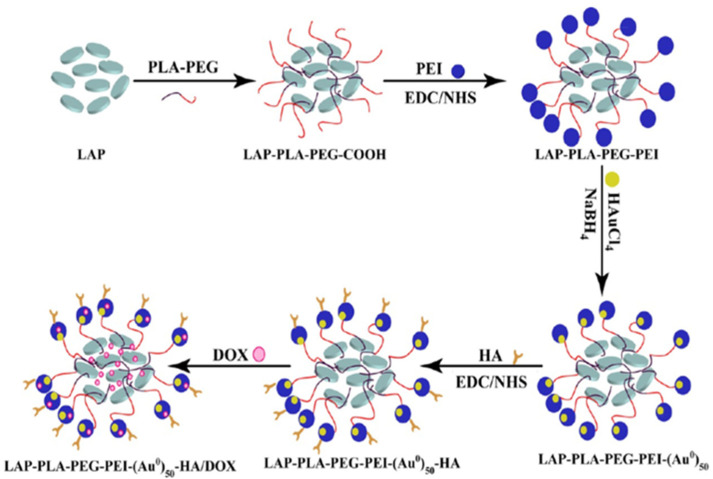
Schematic to obtain LAP-PLA-PEG-PEI-Au-HA/DOX. Reproduced from [62].

A multifunctional theranostic nanoplatform, comprised of laponite, polylactic acid, polyethylene glycol, polyethylenimine, Au and an HA system loaded with DOX drug (LAP-PLA-PEG-PEI--Au-HA/DOX), permits targeted chemotherapy and CT imaging of tumors. These hybrids have high loading efficiency of DOX at 91.0 ± 1.8% and pH-sensitive sustained release. In vitro experiments show that the designed hybrids can selectively deliver to CD44-overexpressing cancer cells, inhibit cancer cell proliferation, and enhance CT imaging. In vivo experiments demonstrate that hybrids can function as targeted contrast agents for CT imaging and effectively suppress tumor growth with reduced side effects [62]. The overall scheme to obtain LAP-PLA-PEG-PEI-Au-HA/DOX multifunctional theranostic nanoplatforms is represented in Figure 3.

In another work, Zhou et al. designed an integrated multifunctional nanoplatform of Au nanorods (NRs), mesoporous silica, HA and Arginylglycylaspartic acid (Au-NRs-mSiO_2_-HA-RGD) for dual-targeted chemo-photothermal therapy. The researchers tested the nanoplatform using DOX, a model drug, to evaluate its drug loading, in vitro drug release profiles, and effects on cells. They found that the nanoplatform demonstrated a favorable photothermal effect and could load drugs at a high capacity of around 20.16%. The additional experimental data examined cellular uptake studies which showed that the Au-NRs-mSiO_2_-HA-RGD nanoplatform could be targeted to ovarian cancer cells through dual mechanisms involving endocytosis mediated by CD44 and integrin receptors [63].

Other parts of the work specify the treatment for cancer stem cells (CSCs) in triple-negative breast cancer (TNBC). To precisely target CD44 receptor-overexpressing cells, together with CSCs, a pentameric nanocomplex (PNC), comprising Au-NPs and DOX conjugated to thiolated HA and PEG, DNA CD44 aptamer, was utilized. At a pH of 4.7, and in the presence of 10 mM glutathione, the most significant in vitro drug release occurred after 8 h. The PNC was nearly ten times more potent when compared with DOX alone [64].

To achieve successful combined photothermal chemotherapy, it is essential to ensure that the photothermal agent is delivered specifically to the tumor and the chemotherapeutic drug is released in a controlled manner. To address these objectives in a single study, a novel nanoplatform called Au-NRs-HA-FA, which incorporates Au-NRs, HA, and FA was developed for breast cancers. The nanoplatform can chemically load DOX through a pH-sensitive hydrazone linkage with around 7.1 wt.% of DOX loading. The designed nanoplatform proved to have good biocompatibility. The nanoplatform decorated with FA showed a notably higher capacity to deliver Au-NRs and DOX to MCF-7 cells through folate receptor-mediated endocytosis. This approach efficiently induced cell apoptosis under NIR irradiation. In vivo experiments showed that the combination of photothermal therapy and chemotherapy resulted in the complete elimination of tumors without causing severe side effects to normal tissues [65].

In another report, the highly potent cytotoxic agent SN38 was conjugated to HA, which was then deposited on the surface of Au-NPs through electrostatic interactions. A loading capacity for SN38 loading of 17.4% was observed and in vitro release studies showed that drug release under acidic conditions was faster when compared with physiological pH environments. The cytotoxicity study on MUC1 positive HT29, SW480 colon cancer cells and MUC1 negative CHO cells indicated that the designed potent hybrids had a higher toxicity on HT29 and SW480 cell lines than on CHO cells [66].

A new approach was developed to synthesize hollow silica nanoparticles (HSNs) containing Au nanocomposites (Au-HSNs) without the use of surfactants, which advances their photothermal properties. The even distribution of Au-NPs in the HSN and presence of a dopamine-hyaluronate (DA-HA) coating on Au-HSN was verified. Under near-infrared irradiation (NIR), the Au-HSN/DA-HA exposed exceptional endocytosis in cancer cells without inducing cytotoxicity [67].

In another study, a novel strategy was discovered to deliver cisplatin selectively to tumors by conjugating it to Au-NPs coated with HA, and to enhance its therapeutic efficacy using laser treatment. In vitro studies demonstrated that the designed systems were more cytotoxic than free cisplatin, and in vivo experiments showed significant antitumor efficacy when combined with near-infrared laser treatment [68].

Zhang et al. reported that Poly (glycidyl methacrylate) (PGMA) microspheres, Au-NPs, and HA produced nanocomposites for precise photothermal application. The PGMA microspheres were obtained using emulsifier-free emulsion polymerization followed by amination, and Au seeds were adsorbed via chelation to obtain Au-PGMA. Further, to target cancer cells specifically and reduce side effects in normal cells, HA was conjugated on the surface of Au-PGMA. The Au-PGMA-HA displayed superior selective targeting toward cancer cells and excellent photothermal outcomes, resulting in three times the therapeutic effectiveness against cancer cells when compared to normal cells [69].

Overcoming multidrug resistance (MDR) in cancer therapy is a significant challenge. To address this issue, a non-viral gene delivery system using HA-conjugated and PEI-modified PEGylated Au nanocages (Au-NCs) loaded with microRNA-21 inhibitor (anti-miR-21) was developed to enhance the efficacy of DOX. In vitro studies showed the HA/anti-miR-21/PP-Au-NC system increased intracellular DOX accumulation and sensitized DOX-resistant HCC cells (HepG2/ADR cells) by upregulating PTEN protein expression and downregulating P-gp protein expression. Additionally, mild NIR led to hyperthermia of the HA/PP-Au-NCs, further improving the therapeutic effects. Moreover, the HA/anti-miR-21/PP-Au-NCs system showed good biocompatibility, highlighting its significant role as a new strategy for cancer treatment with MDR [70].

The effective exploitation of the EPR result of tumors requires careful consideration of NP sizes. Larger particles have good retention but poor penetration while smaller ones have the opposite effect. Size-reducible NPs have been developed to address this issue, although the primary size and complex tumor microenvironment continue to limit their distribution. To overcome these challenges, size-reducible nanoplatforms using hyaluronidase-degradable HA and cationic bovine serum albumin (CBSA) protected Au-NCs have been obtained. The ratio of HA to Au-NC-CBSA, Au-NC-CBSA-HA can be adjusted and varying initial sizes designed so as to evaluate the pharmacokinetic profiles and tumor-targeting efficiencies. Furthermore, the Au-NC-CBSA-HA platform. with a size of 200 nm, can be utilized to load paclitaxel (PTX) and indocyanine green (ICG) for chemo-photothermal therapy, as well as nitric oxide (NO) to enhance drug delivery and modulate the tumor microenvironment. The final construct, Au-NC-CBSA-PTX-ICG-HA-NO_3_, exhibited a size-reducible property triggered by hyaluronidase and high accumulation with homogenous intra-tumor distribution. The construct was successful in reducing tumor growth by 95.3% and inhibiting the growth of lung metastasis by 88.4%, demonstrating its potential as an effective strategy for improved antitumor therapy [71].

**Figure 4:**
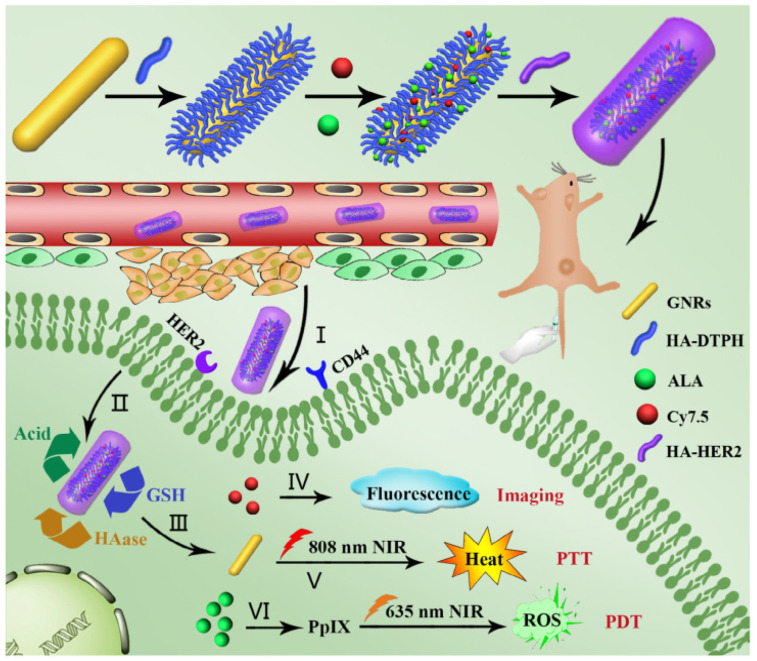
Scheme and respective mechanisms for Au-NR-HA/ALA/Cy7.5-HER2. nanoplatforms. Reproduced from [72].

In other research, a versatile nanoplatform was fabricated by functionalizing gold nanorods (Au-NRs) with HA (containing hydrazide and thiol moieties). Then, 5-aminolevulinic acid (ALA), Cy7.5 and anti-HER2 antibody were chemically conjugated onto the HA moiety for PDT, fluorescence imaging and active targeting, respectively. This nanoplatform remarkably enhanced the cellular uptake of Au-NR-HA/ALA/Cy7.5-HER2 in vitro. The scheme and respective mechanisms are displayed in Figure 4. In the presence of NIR irradiation, MCF-7 cells were efficiently killed by a combination of PDT and PTT. This nanoplatform could be specifically delivered to tumor tissues with an accumulation ratio of 12.8%. This specific PDT/PTT nanoplatform-based treatment completely eliminated tumors without obvious side effects, revealing impressive potential in cancer therapy [72].

Another study designated the development of a light-responsive drug delivery system based on host-guest chemistry. This system consists of a gold nanorod (Au-NR) that generates plasmonic heat upon NIR exposure, and a layer of HA immobilized to the Au-NR via functionalization with the macrocycle. It further released a retinoic acid (RA) derivative, a molecule important in tissue development, homeostasis, and cancer treatment. The formulation and the bioactivity of the released RA was demonstrated in a reporter cell line expressing luciferase controlled by the RA receptor [73].

The dual combination of chemotherapy and photothermal therapy has proved a promising approach for treating cancer. To achieve a multifunctional conjugated system, oxidized HA decorated dihydroxyphenyl/hydrazide biofunctionalized hydroxyethyl chitosan (DHHC)-Au-NR is used. The DOX was loaded onto the conjugate resulting in a drug loading content of 5.1%. The DOX-loaded multifunctional conjugated system showed good stability in neutral aqueous solutions and had pH-responsive drug release. In vitro studies demonstrated that the conjugate was efficiently internalized by MCF-7 cells and had synergistic therapeutic effects [74].

Another study reported on a nanosystem which is highly responsive to multiple stimuli and can deeply penetrate tissues, co-delivering poly(amidoamine) (PAMAM) stabilized AuNPs and pH-responsive DOX prodrug (PD conjugate) incorporated into a HA-based nanoshell. By leveraging the different properties of the PD, Au-NPs, and HA nanoshell, its feasibility was demonstrated both in vitro and in vivo, achieving remarkable intratumoral penetration and a synergistic radio-chemotherapeutic effect [75].

**Figure 5:**
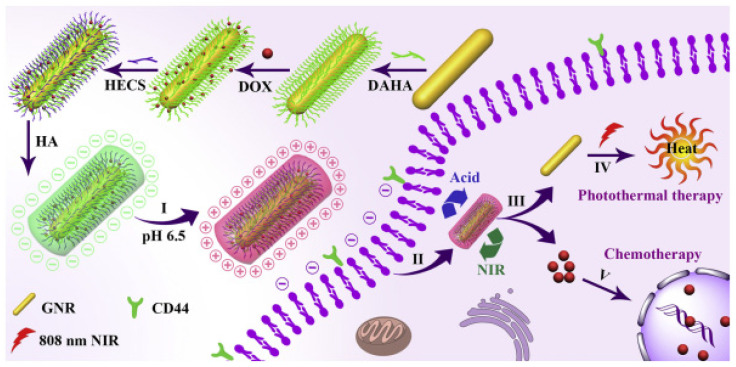
Scheme to produce AU-NR-DAHA-DOX-HECS-HA and the therapeutic mechanisms. Reproduced from [76].

It is important to note that combining multiple therapeutic modalities that utilize distinct mechanisms for eliminating tumors has become an encouraging approach for treating cancer. Focusing on such aspects, an innovative platform was developed using chemo-photothermal therapy of breast cancer, which utilizes an aldehyde/catechol-functionalized HA (DAHA) and hydroxyethyl chitosan (HECS) decorated Au-NR. The resulting nanoplatform proved to have 4.1% of DOX content and exhibited pH/NIR drug release behaviors. The designed nanoplatform was effectively taken up by MCF-7 breast cancer cells and displayed superior efficacy in eliminating cancer cells when compared to the individual therapeutic modalities [76]. The scheme involved in producing AU-NR-DAHA-DOX-HECS-HA and the therapeutic mechanisms are represented in Figure 5.

Chen et al. developed a new drug carrier based on porous silica (pSiO_2_), with a gatekeeping system composed of an HA layer and Au-NPs. Whilst pSiO_2_ served as the drug carrier, the HA and Au NPs acted as the gatekeepers to control drug release. The amoxicillin (Amox) loading content was 18.2% and the release rate was regulated by redox-induced breaking of S-Au bonds and enzymatic degradation. The pSiO_2_-Au/HA composite exhibited remarkable photothermal conversion efficiency and repeatability [77].

**Figure 6:**
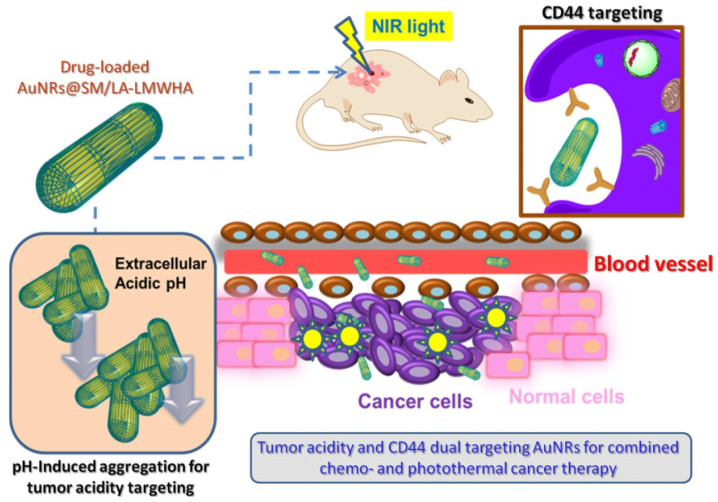
Tumor acidity and CD44 dual targeting process by loading DOX drug for combined therapy. Reproduced from [78].

Another part of the research work explored the potential of HA-coated Au-NRs for combined chemo and photothermal cancer therapy by targeting both tumor acidity and CD44, as shown in Figure 6. To achieve this pH-induced aggregation and Au-NR coating, low molecular weight hyaluronic acid (LMWHA) was conjugated with pH-sensitive groups and lipoic acid (LA). The modified LMWHA’s pH sensitivity could be adjusted by changing the pKa values of the pH-sensitive groups. The biocompatibility of the coated Au-NRs was significantly enhanced. The LMWHA-coated Au-NRs could progressively aggregate under minor acidic conditions, promoting accumulation at tumor sites and the Au-NRs provided excellent photothermal ability. Loading DOX on the nanosystem showed 5.0% loading capacity and enhanced cancer cell-killing and tumor growth inhibiting abilities [78].

The focus of another research investigation involved development of gold nanochains (Au-NCs) with worm-like nanostructures as a theranostic system for efficient photodynamic therapy (PDT) under light irradiation. To achieve this, citrate-stabilized Au-NPs were assembled using HA and hydrocaffeic acid (HA-HCA) conjugates as templates. The photosensitizers (PSs), and tumor-targeting ligands were integrated onto the surfaces of the Au-NCs and were highly selective, showing notable phototoxicity, even at low PS concentrations, when exposed to laser irradiation [79].

Generally, the Au-core mesoporous silica shell (Au-MSS) offers a versatile and promising approach in cancer photothermal therapy. Nonetheless, restricted half-life in the bloodstream and the low specificity towards tumor tissue have constrained the potential use in further applications. To address such issues, in this study, D-α-Tocopherol polyethylene glycol 1000 succinate (TPGS) and HA were conjugated to improve the biological performance of Au-MSS. Furthermore, the Au-MSS functionalization improved the hemocompatibility and selectivity of the nanomaterial towards cancer cells. Moreover, it successfully induced the death of HeLa cancer cells through an on-demand photothermal effect [80].

Li et al. reported on a small-sized nanocomposite for cancer therapy and diagnosis. The nanocomposite was composed of chlorine e6 (Ce6) integrated Au nanoclusters (NCs) (Au:Ce6 NCs), HA, DOX, and FA. The FA conjugation of the nanocomposite further enhanced the cellular selectively target, while its pH-responsive nature controlled the release of DOX for tumor chemotherapy. The nanocomposite displayed good biocompatibility, stability, and loading capacity for Ce6 and DOX were 11.3 and 10.00%, respectively. The combination of chemotherapy and PDT in the nanocomposite resulted in significant cancer cell death upon exposure to laser irradiation [81].

**Figure 7:**
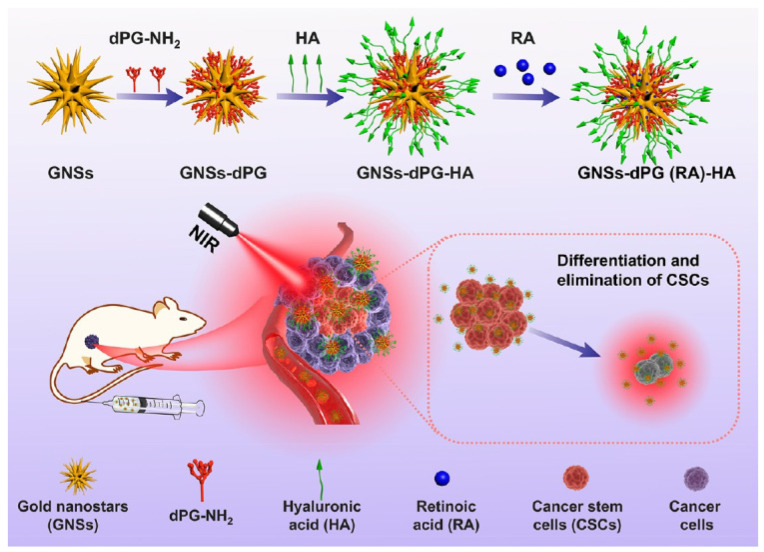
Scheme for RA-loaded GNSs-dPG for targeted photothermal therapy. Reproduced from [82].

The presence of cancer stem cells (CSCs) represents a foremost challenge to the effectiveness of existing cancer treatments, as non-CSCs can instinctively convert into CSCs, leading to treatment failure and tumor recurrence. Therefore, developing effective strategies to eradicate CSCs is crucial. To solve these issues, a CSC-specific, RA-loaded Au-NS with dendritic polyglycerol (Au-NSs-dPG) nanoplatform was developed for efficient CSC eradication. The designed system exhibited excellent biocompatibility and effective CSC-specific multivalent targeting through HA decoration on the bioinert dPG’s multiple attachment sites. Furthermore, RA-induced CSC differentiation combined with PTT yielded high therapeutic efficacy in a synergistic inhibitory manner, suppressing breast CSCs and tumor growth. Moreover, the expression of stemness genes and CSC tumorsphere development were notably reduced. In vivo, the nanoplatform effectively eliminated tumor growth and CSCs, indicating higher anticancer activity and effective CSCs suppression [82]. The scheme for RA loaded GNSs-dPG for targeted photothermal therapy is shown in Figure 7.

**Figure 8:**
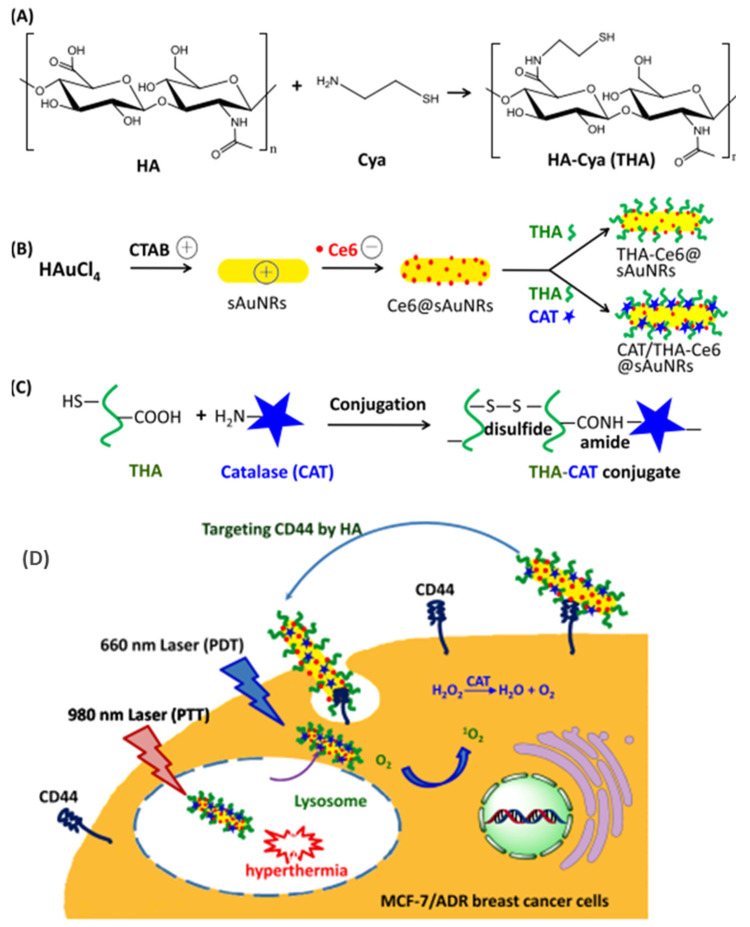
(**A**) Au-Cya, (**B**) CAT/THA-Ce6-Au-NRs, (**C**) CAT-THA (**D**) Scheme displaying cellular uptake of CAT/THA-Ce6-Au-NRs with prospects of combined therapy. Reproduced from [83].

In recent work, Ce6-adsorbed small Au nanorods (Ce6-sAu-NRs), that can activate photothermal and photodynamic effects through NIR/visible light, were produced. The Ce6-sAu-NRs were decorated with biological macromolecules, such as thiolated HA and catalase (CAT), to enable targeting of CD44 and self-supply of O_2_. This platform unveiled visible-light absorption at 668 nm and robust NIR absorption at 800–1000 nm. The CAT/THA-Ce6-sAu-NRs were able to easily accumulate in CD44-overexpressing MDR breast cancer cells via CD44-HA recognition. Additionally, CAT catalyzed the decomposition of endogenous H_2_O_2_ to generate O_2_, thus mitigating hypoxia and ensuring high PDT efficiency [83]. The scheme and the cellular uptake of CAT/THA-Ce6-Au-NRs with prospects of combined therapy are represented in Figure 8.

A very recent report involves the advance of a pH/NIR dual stimulus-responsive delivery system fabricated using polydopamine (PDA) mediated Au with HA, loaded with DOX drug (PDA-Au-HA/DOX), having loading content of around 10.00 wt.%, to targets tumors. The developed system is coated with HA, which endows it with tumor-targeting capabilities by binding specifically to the CD44 receptor, which is overexpressed in various tumor cells. The PDA and Au nanoshells exhibited excellent photothermal performance under NIR irradiation, destroying tumor cells and accelerating the release of DOX [84]. Overall, the core materials used, other substituents and key outcomes are summarized in Table 1.

### 6.2. HA-Modified Ag-M-NPs and Other Substituents

In 2018, HA and AgNP combinations which exploit the electrostatic interaction between negatively charged HA molecules and positively charged AgNPs, followed by ultrasonication-induced assembly, were introduced. The cell line studies for HA-Ag-NPs exhibited anti-leukemic activity via ROS overproduction compared to AgNPs alone. Furthermore, the outcomes indicated that HA-Ag-NPs significantly inhibited leukemia cell viability by inducing apoptosis via specific binding of HA with CD44 receptors that were overexpressed on the cell surface. Therefore, HA-Ag-NPs represent a novel approach for leukemia treatment that takes advantage of altered redox conditions in cancer cells and reduces systemic toxicity. These findings provide valuable insights into the design and improvement of leukemia-specific chemotherapy [85].

Another study involved using HA as a gel-forming agent, and Amanita muscaria extract was utilized as the capping agent during the synthesis of Ag and ultra-small iron oxide to obtain Fe-Ag-NPs for synergistic anticancer properties. The potential of the HA/Fe-Ag gel for localized cancer treatment was demonstrated through cytotoxicity studies conducted on both 2D and 3D HeLa cell cultures. The gel formulation utilized HA as a gelling agent and it was observed that HA improved the transportation of the active components within HeLa spheroids, thereby enhancing their effectiveness. These findings suggest that the HA/Fe-Ag NPs have potential as a beneficial approach for cancer treatment [86].

In order to overcome the limitations of “single-strategy” therapy in real body internal environments, Liu et al. utilized HA-modified Ag-S-nitrosothiol core-shell NPs using (EGDMA, TEOS) for synergistic therapy, based on a combination of PTT and nitric oxide (NO)-based chemotherapy. Under NIR, the Ag core generated cytotoxic heat leading to cancer cell apoptosis. In addition, the S-nitrosothiol polymeric shells responded to NIR and heat by releasing free NO at high concentration, which induced NO-based chemotherapy. The efficacy of the photothermal and NO-based chemical synergistic therapy in targeting tumors was demonstrated through both in vitro cytotoxicity assays and in vivo experiments conducted on mice with tumors [87]. Overall, the core materials used, other substituents and key outcomes are summarized in Table 2.

### 6.3. HA-Modified Pt /Pd-M-NPs and Other Substituents

The use of targeted photothermal therapy (PTT) in cancer treatment can enhance therapeutic outcomes while minimizing side effects. Nevertheless, incorporating additional functionality comes at the cost of increased synthetic steps, toxicity issues, and complex in vivo behavior effects. To address such difficulties, the one-pot method is used to produce HA/Pt tumor-targeted systems. Further, in vitro experiments validated that CD44-overexpressing cancer cells were internalized more effectively than non-targeted alginate acid-Pt nanoparticles (AA/Pt). Similarly, in in-vivo studies HA/Pt accumulated more in CD44-overexpressing tumors than AA/Pt, and proved to have superior efficacy in inhibiting tumor growth through PTT [88].

It is important to mention that developing a nanoplatform that can effectively target hypoxic tumors using PDT is critical in contemporary cancer research. Thus, in this work a ROS-generator, called HA-modified Pt NPs/carbon dots-loaded mesoporous silica (HA-PCD), was designed. The HA-PCD is composed of Pt NPs and carbon dot (CD)-loaded dendritic mesoporous silica nanoparticles (DMSNs), further modified with HA. When exposed to 635 nm laser irradiation, HA-PCD produces ^1^O_2_ due to the involvement of CDs photosensitizers. The loaded Pt NPs enhances photodynamic therapy under hypoxic conditions by producing oxygen via catalase-mimicking activity. Moreover, it produces OH and O_2_− for catalytic therapy, due to peroxidase and oxidase-mimicking actions [89].

**Figure 9:**
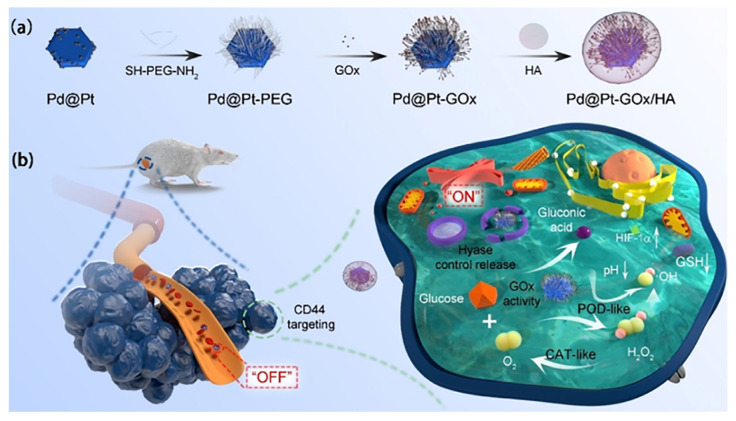
Synthetic process involved in producing (**a**) Nanozyme assisted starving improved chemo-dynamic therapy and (**b**) Cellular mechanisms for Pd-Pt-GOx/HA. Reproduced from [90].

Another work described novel multifunctional drug delivery systems developed using, Pt, Pd, glucose oxidase (GO) and HA to obtain Pd-Pt-GO/HA, which addressed the low efficiency and potential side effects to normal tissues associated with GO-mediated starvation therapy. The Pd-Pt-GOx/HA system specifically targets CD44-overexpressed cancer cells and possesses intracellular Hyase-responsive GO, catalase (CAT), and peroxidase (POD)-like activities as well as glutathione (GSH) oxidation capacity, significantly enhancing therapeutic efficacy and biosafety. The differential uptake of Pd-Pt-GO/HA by cancer cells and normal cells demonstrated that the reactive oxygen species (ROS) induced cell apoptosis. Furthermore, in vivo experiments demonstrated the excellent treatment efficacy of Pd-Pt-GO/HA on 4T1 and h22-tumor-bearing mouse models [90]. The representative scheme and cellular mechanisms are shown in Figure 9.

Zhang et al. developed a nanosystem for photothermal therapy (PTT) and antioxidant therapy by constructing Pd-Se-HA nanosystems. The selenium (Se) NPs and Pd NPs were integrated into the core-shell structure, where the Pd NPs showed photothermal effects. Further, HA was bonded to the surface of the nanosystem to provide targeting functions and to form Pd-Se-HA nanosystems. In vitro studies demonstrated good photothermal effect, -OH scavenging ability, effective inhibition of macrophage infiltration, ROS production, and cytokine-mediated inflammation. In addition, after 15 days of treatment, the Pd-Se-HA almost completely stopped the inflammatory response in the joints of mice with an induced RA model and stopped joint degradation [91].

To develop new inorganic sonosensitizers for sonodynamic therapy (SDT), two primary goals should be noticed, such as increase in the formation of ROS and decrease in ROS elimination. In this work, new SDT systems were designed by using unique combinations of barium titanate oxide NPs, Pd, manganese dioxide and HA to produce BTO-Pd-MnO_2_-HA. The deposition of Pd NPs creates Schottky junctions that separate electron-hole pairs, raising the competence of toxic ROS production during SDT. The MnO_2_ degrades within the tumor microenvironment (TME), and the Mn^2+^ ions catalyze the Fenton-like reaction generating •OH from H_2_O_2_. The BTO-Pd-MnO_2_-HA incessantly consumes glutathione (GSH) and produces O_2_, which improves SDT and chemodynamic therapy (CDT) efficiency. The BTO-Pd-MnO_2_-HA offers a multistep. improved SDT process that is activated by TME decomposition, targeted by HA, and amplified by Pd depositions [92]. To conclude, the core materials used, other substituents and key outcomes are summarized in Table 3.

## 7. Application of HA-Modified Non-noble M-NPs and Other Substituents in Various Cancer Therapy

### 7.1. HA-Moadified Magnetic-M-NPs and Other Substituents

A water-based two-step method was used to produce hybrid combinations of superparamagnetic iron oxide NPs, chitosan and HA loaded with curcumin drug (SPION-CCh-HA-Cur). The SPIONs have a core size of slightly above 10 nm and the designed hybrid systems exhibit high magnetic properties, making them suitable for use as MRI contrast agents. Furthermore, biological studies showed that hybrid systems can be easily internalized into cells and did not exhibit cytotoxicity at the tested concentration [93].

In another work, HA-modified SPIONs were developed and placed upon NIR. The HA-SPIONs generate heat rapidly and in-vitro studies showed that HA-SPIONs revealed noteworthy explicit cellular uptake and accumulation in CD44 HA receptor-overexpressing MDA-MB-231 cells. Furthermore, improved magnetic resonance imaging (MRI) and photothermal ablation, both in vitro and in vivo, demonstrated substantial photothermal effects specifically targeting CD44 HA receptor-overexpressing breast cancer [94].

Other work reported conjugation of hyaluronic acid (HA) and bovine serum albumin (BSA)-modified zinc copper indium sulfide quantum dots (ZCIS QDs) onto the surface of polyethyleneimine (PEI)-coated iron oxide-Prussian blue NPs (Fe_3_O_4_-PB). The resulting Fe_3_O_4_-PB-HA-BSA-ZCIS QDs, denoted as FPPBH, demonstrated good biocompatibility and better adsorption in the NIR region. In vitro studies revealed specific uptake of FPPBH by CD44 overexpressed HeLa cells when an external magnetic field was applied. In vivo NIR fluorescence and magnetic resonance imaging demonstrated the high accumulation of FPPBH at the tumor site due to the exceptional CD44 receptor/magnetic targeting ability. The tumor was successfully ablated in nude mice after intravenous FPPBH injection and treatment with an external magnetic field, which led to a tumor growth inhibition rate of more than 89.95% when the tumor was exposed to NIR light [95].

In another study, in order to treat hepatocellular carcinoma, a dual system of HA and DOX was synthesized and subsequently conjugated with amine-modified Fe-NPs. The resulting hybrid system possessed good water dispersibility, superparamagnetic properties, and high magnetic relaxivity. In addition, the hybrid system proved to have notable cellular uptake and accumulation in HepG2 cells, a type of human liver cancer cells, which is believed to be due to the definite role of HA. Furthermore, in vitro studies revealed that the release of DOX from the hybrid system was markedly accelerated under mild acidic conditions (pH 5.0–6.0), which is ideal for effective chemotherapy. Lastly, the in vivo antitumor efficacy of these hybrid systems was demonstrated in mice, confirming their substantial therapeutic potentials [96].

Another significant investigation reported that a novel magnetic nanovehicle was developed using HA conjugated iron oxide (IONPs) for targeted delivery of chemotherapy drugs to tumor areas with external magnetic field guidance. Moreover, the IONPs were capable of carrying homocamptothecin (HCPT) model drug, and 75% of HCPT was encapsulated in the HA-IONPs. The in vitro and in vivo experiments demonstrated remarkable magnetic tumor targeting and effective tumor cell ablation. Notably, no systemic toxicity was observed, highlighting the potential clinical translatability of the designed nanovehicle as a magnetic field responsive platforms for targeted delivery applications [97].

Zheng et al. developed nanoplatforms comprised of HA-SPION micelles, loaded with docetaxel (DTX) drug, with a loading efficiency of 10.9%. The results of cellular uptake studies demonstrated that MDA-MB-231 cells were internalized via CD44 receptor-mediated endocytosis, due to the existence of a magnetic field and evidenced good MRI potential. Additionally, the micelles achieved superior localized photothermal ablation attainment in MDA-MB-231 cells, demonstrating their potential as effective photo-absorbers in photothermal therapy [98].

**Figure 10:**
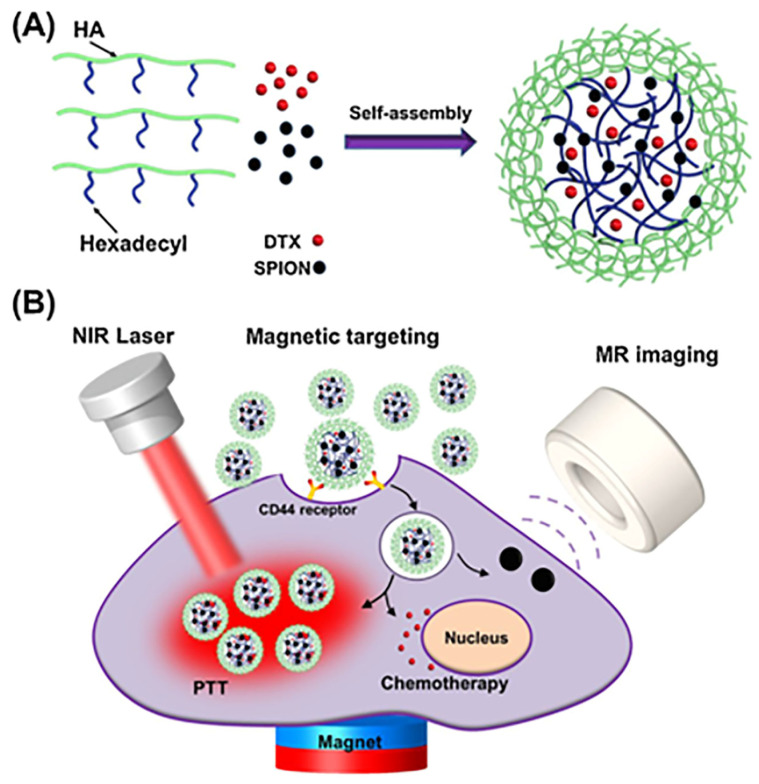
(**A**) Scheme showing micelle loaded with DTX drug and (**B**) Dual tumor targeted therapies. Reproduced from [98].

The overall scheme to obtain micelle-loaded DTX drug and the dual tumor targeted therapies are shown in Figure 10.

A new nanoplatform with synergistic chemo-photothermal therapy was designed using magnetic polydopamine (MPDA), HA-MTX for PTT. The nanoplatform proved to have excellent biocompatibility and photothermal conversion efficiency suggesting potential for photothermal therapy, as well as improved cellular uptake and drug release. Additional studies conducted both in vitro and in vivo showed that MPDA-HAMTX with DOX added had preferential tumor accumulation, improved specificity to target tumor cells, pH- and laser-responsive release, and a high tumor cell-killing efficiency [99].

Other research studies revealed HA-PEGylated magnetic nanoparticles (HA-PEG-MNPs) prolonged the circulation time of mitoxantrone (MTX) and targeted specific tumor cells. The MTX loading efficiency was around 87.7%, the release of MTX from HA-PEG-MNPs was mainly inhibited by amide linkages, and HA-PEG-MNPs remained stable in physiological conditions for up to 8 days. In addition, HA-PEG-MNPs could bind to the receptor-binding site and internalize into tumor cells, proving significant induction of apoptosis in MDA-MB-231 cell lines [100].

Another report described utilization of FePt alloy nanoparticles with precise sizing, which were subsequently treated with (3-Aminopropyl) triethoxysilane (APTES) to modify their surfaces. Through a pH-sensitive hydrazone bonding process, lenalidomide (LND) was covalently bonded to FePt-NH_2_ and validated with an LND loading efficiency of 6.3%, while APTES amino groups were used to attach HA. Additionally, it was conjugated with lactoferrin (Lf)-bearing carboxylic groups on the HA, which led to the development of surface-modified pH-sensitive alloy-drug nanoconjugates known as SPANs. These SPANs demonstrated exceptional heat generation upon exposure to magnetic fields and NIR. Due to the leaching of Fe and Pt contents, SPANs were capable of generating ROS in the U87MG cell line, thereby enhancing their therapeutic effects. The in vivo results confirmed enhanced uptake of SPANs in the brain after intranasal administration with improved nasal and mucus penetration due to the presence of Lf [101].

Luo et al. developed stable and cytocompatible HA-Fe_3_O_4_ NPs using PEI and mPEG for targeted MRI of pancreatic cancer. The cellular uptake analysis results showed that MIAPaCa-2 cells, which overexpress the CD44 receptor, were specifically internalized by HA-Fe_3_O_4_ NPs. Therefore, the developed system could serve as an effective nanoprobe for the MRI of pancreatic cancer cells [102].

Another work involved a solvothermal method to create Fe_3_O_4_ NPs coated with porous carbon (PC), followed by amine terminated groups which were subsequently modified with HA for targeted tumor treatment. The designed system unveiled exceptional biocompatibility and efficient photothermal transformation capability, and the porous structure allowed for a high DOX drug loading capacity of around 27% and intelligent drug release, making it a multifunctional nanodrug delivery system. In vivo T_2_-weighted MR imaging displayed the accumulation of nanocarriers in the tumor. Both in vitro and in vivo studies were conducted to confirm the efficacy of the system [103].

In other work layered double hydroxides (LDHs) conjugated Fe_3_O_4_ tagged with HA to load DOX were reported on, for improved T1-weighted MR imaging and chemotherapy of cancer cells that overexpress CD44 receptors. The reported LDH-Fe_3_O_4_-HA demonstrated a 10-fold increase in r1 relaxivity compared to Fe_3_O_4_ NPs and proved to have57.65% DOX loading capacity. The designed LDH-Fe_3_O_4_-HA demonstrated pH-responsive release behavior, and showed targeted tumor inhibition effect in vitro. The in vivo result indicated improved tumor penetration and significantly enhanced MR imaging ability [104].

In another study, through a simple process, dual-stage carcinoma cell-targeting systems for DOX delivery were witnessed. The interaction between the coated phosphatidylcholine PC/HA surface and embedded DOX-Fe_3_O_4_ had a significant impact with good antitumor efficacy for MDR cancer therapy with minimal cardiotoxicity. Furthermore, PC/HA-DOX-Fe_3_O_4_ was able to deliver DOX to a xenograft tumor, and could concentrate into the tumor cells in in vivo studies [59].

Soleymani et al. substantiated a simple one-pot system for synthesizing HA-coated Fe_3_O_4_ with an appropriate size for magnetic hyperthermia therapy and targeted CD44 overexpressing cancer cells. The deigned system unveiled excellent colloidal stability and low cytotoxicity towards L929 cells. Further, Fe_3_O_4_-HA NPs preferentially targeted MDA-MB-231 cells with a 4-fold higher uptake than L929 cells. Additionally, the heat generation capability of Fe_3_O_4_-HA NPs under different permissible magnetic fields indicated an intrinsic loss power (ILP) value of Fe_3_O_4_-HA NPs of about 3.5 nHm^2^/kg, which was about 25-fold higher when compared with bare Fe_3_O_4_ NPs [105].

Another study explored multifunctional nanocarriers for cancer therapy using a combination of magnetic and photothermal therapies. The cisplatin-loaded NIR-responsive PLGA magnetic nanoparticles were coated with HA and labelled as HA/PMNPc. The PMNPc, encapsulating oleic (OA) modified iron oxide nagnetic NPs (IOMNP), allowed for magnetic targeted drug delivery. By varying the amount of cisplatin it was possible to obtain loading capacity differences from 5.3 to 18.00%. The incorporation of HA to PMNPc resulted in a much higher intracellular uptake efficiency and active targeting of U87 cancer cells. In a xenograft tumor model in nude mice, treatment with HA/PMNPc via tail vein injection, resulted in the lowest tumor growth rate. The dual-targeting capability of HA/PMNPc makes it a promising multifunctional platform for effective cancer therapy against U87 glioblastoma cells [106].

One of the main drawbacks involved in chemotherapy is inadequate adhesion of drugs in tumors, leading to the failure of cancer cell growth prevention. To enhance such drawbacks, a highly efficient nanocarrier, designed by modifying iron oxide nanoparticles (IONPs) with a tumor-targeting peptide c(RGDyK) and hyaluronidase (HAase) on the surface, exhibited DOX loading capacity of around 21.7%. The resulting nanocomplex bound to integrin αvβ3 to target the tumor and penetrated intensely into the tumors by degrading the highly expressed HA in the tumor extracellular matrix (ECM). In vitro, c(RGDyK)-HAase-IONP carrying DOX showed good biostability and a preferred drug release profile at low pH. After intravenous injection in MC38 tumor-bearing mice model, c(RGDyK)-HAase-IONP exhibited a 2.5 times higher tumor-targeting effect [107].

In another research work, a magnetic nanocarrier sensitive to pH was developed for the delivery of DOX, through grafting HA/β-cyclodextrin onto Fe_3_O_4_ magnetic nanoparticles. In-vitro release behavior for DOX was evaluated at two different pH levels: simulated human blood fluid (pH = 7.4) and simulated cancer fluid (pH = 5.6). Strong pH dependence was pbserved. The nanocarrier’s pH-sensitive release performance resulted in a higher DOX release at pH = 5.6 (92.43%; 48 h) than at pH = 7.4 (77.05%; 48 h). Moreover, the results demonstrated that the DOX release mechanism from the nanocarrier was guided by Fickian diffusion kinetics [108]. Overall, the core materials used, other substituents and key outcomes are summarized in Table 4.

### 7.2. HA-Modified Zn-M-NPs and Other Substituents

**Figure 11:**
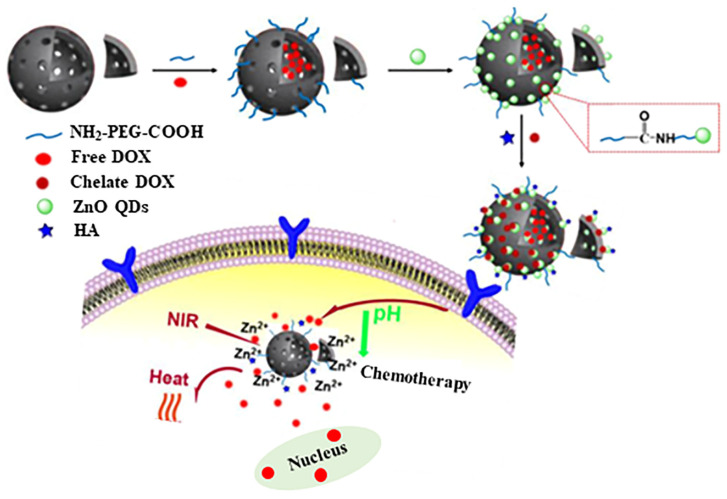
Scheme illustrating the production of the OMCN nanosystem for targeted cellular uptake. Reproduced from [109].

In order to enhance therapeutic effectiveness, a new system was designed using PEG-modified oxidized mesoporous carbon nanospheres (OMCNPs) loaded with DOX drug. To target lung cancer cells, the OMCNPs were modified with HA. Furthermore, zinc oxide quantum dots (ZnO QDs) were added to not only cap the OMCNP, but also to chelate with DOX, resulting in 52% loading content. Upon cellular uptake, the pH-sensitive ZnO lids dissolved to Zn^2+^ in tumor cells, leading to dissociation of the Zn^2+^ DOX complex and controlled release of DOX. The use of the OMCNP-based system can induce hyperthermia and promote the release of DOX when exposed to NIR irradiation. When combined with targeted chemo-photothermal therapy, this approach yielded better results than either single chemotherapy or photothermal therapy alone [109]. The scheme to produce the OMCN nanosystem for targeted cellular uptake is schematically displayed in Figure 11.

In another report, a bioreducible carrier for siRNA delivery, was created by conjugating Zn (II)-dipicolylamine onto HA (HA-DPA(Zn)) to coordinate with siRNAs and create stable formulation in the presence of zinc ions. The siRNA formulated with this carrier was efficiently taken up by U87MG cells and released the incorporated siRNAs in response to reduction signals. In vitro studies demonstrated that siRNA formulated HA (HA-DPA(Zn)) effectively silenced genes with minimal toxicity. It also proved to have a prolonged circulation time in the bloodstream, enhanced accumulation in the tumor site, and remarkable antitumor efficacy in a U87MG tumor-bearing mouse model, without triggering any organ toxicity [110].

Another research study conjugated zinc oxide (ZnO) through a co-precipitation method with HA to obtain (HA-ZnO). The conjugated system was then modified with ginsenoside Rh2, to obtain Rh2-HA-ZnO. The designed Rh2-HA-ZnO exhibited anti-cancer effects on three different cancer cells, namely, A549 lung cancer, HT29 colon cancer, and MCF7 breast cancer cells. Additionally, intracellular ROS were observed in all three cancer cell lines [111].

**Figure 12:**
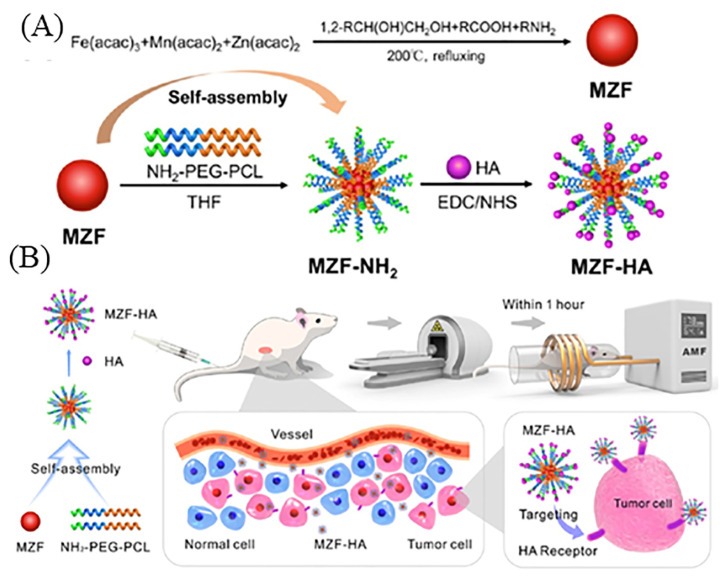
Outline scheme to obtain (**A**) MZF and their surface modification to produce MZF-HA and (**B**) demonstration to use MZF-HA system for targeted cancer therapy. Reproduced from [112].

In the treatment of non-small-cell lung cancer (NSCLC), radiotherapy (RT) is a key approach, but there is a critical need to amplify its negative effects on tumors through the development of new treatment modalities. To address such problems, Wang et al. prepared block copolymer micelles comprised of PEG and polycaprolactone (PEG-PCL) comprising HA manganese and zinc (Mn-Zn) ferrite magnetic nanoparticles (MZF). Furthermore, micelles with HA-modified MZF resulted inMZF-HA for specific targeting of CD44 highly expressing tumor cells, such as A549 (human lung adenocarcinoma cell line). In the A549 subcutaneous tumor xenografts model, the MRI proved the enhancement of MZF-HA in the tumor, and hypoxia immunohistochemistry analysis (IHC) confirmed enhanced tumor oxygenation after HT. Furthermore, there was a 49.6% decrease in tumor volume, compared to a 58.8% increase in the untreated group [112]. The outline scheme to obtain MZF and the surface modification to produce MZF-HA and a demonstration using the MZF-HA system for targeted cancer therapy is shown in Figure 12.

**Figure 13:**
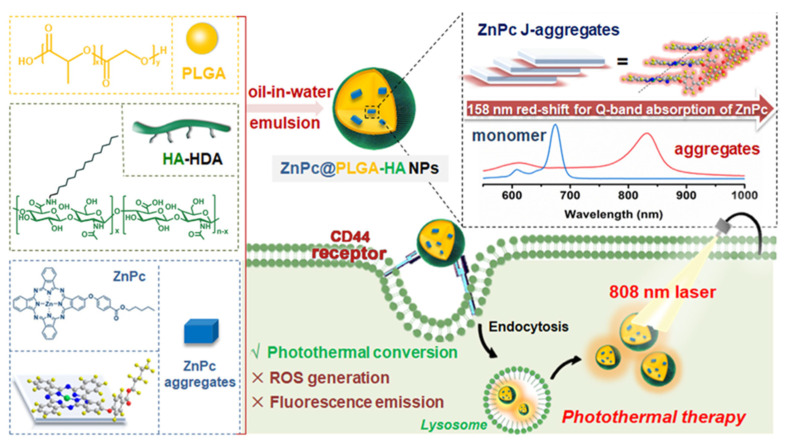
Scheme to obtain ZnPc-PLGA-HA system and their respective mechanisms. Reproduced from [113].

In another work, biodegradable NPs which can target tumors were developed for photothermal therapy (PTT) against human cancer cells that overexpress CD44. The zinc (II) phthalocyanine-based photosensitizer (ZnPc) was loaded to PLGA-HA, and exhibited high stability and good biocompatibility. Upon 808 nm irradiation, the ZnPc-PLGA-HA induced a photothermal effect and promoted cellular uptake by CD44-overexpressed A549 and HT29 cells, leading to enhanced photothermal efficacy. Moreover, the ZnPc-PLGA-HA was able to ablate the tumor of nude mice upon laser irradiation [113]. The scheme to obtain the ZnPc-PLGA-HA system and the respective mechanisms are shown in Figure 13.

### 7.3. HA-Modified Ce-M-NPs and Other Substituents

Despite significant progress in breast cancer treatment, the challenge of addressing the aggressive nature of TNBC is considered a major obstacle. Thus, to deliver a solution, PEI assisted HA tagged with ceria (PEI-HA-Ce) can be used as a therapeutic agent, and has demonstrated significant anticancer effects and is responsible for the generation of ROS for MDA-MB-231 TNBC cells. The designed PEI-HA-Ce demonstrated efficient endocytosis, resulting in MMP loss and successive release of Cyt c from the mitochondria. This led to the activation of caspases-3 and -9, while also decreasing levels of Bcl-2. Treatment with PEI-HA-Ce led to irreversible nuclear chromatin condensation [114].

**Figure 14:**
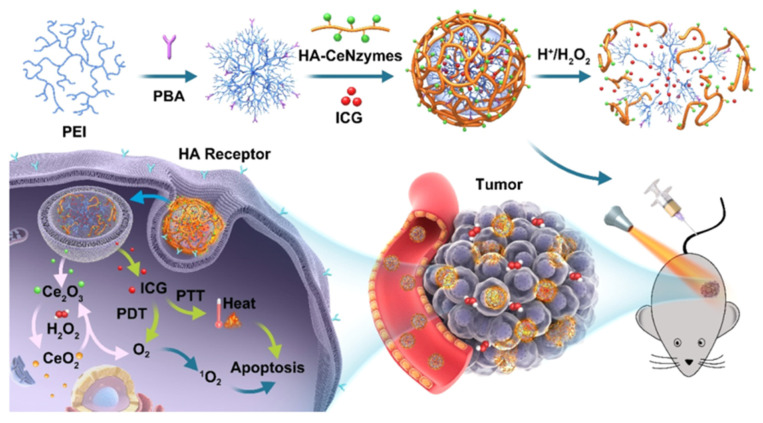
Scheme and the mechanisms involved in utilizing pH sensitive polymer, HA mediated inorganic enzyme and ICG loading to produce ROS. Reproduced from [115].

Other work resulted in a dual-targeted drug delivery system for solid tumors by using a pH-sensitive polymer and an inorganic nanozyme. The core of the PEI polymer was loaded with indocyanine green (ICG) by electrical charge adsorption. Once delivered to the tumor site, the CeO_2_ NPs catalyzed the production of oxygen from hydrogen peroxide through the cycling of cerium valency, thus enhancing both PTT and PDT. This strategy successfully improved the hypoxic microenvironment of the solid tumor. Additional results verified that ICG-PEI-HA/CeO_2_ increased ICG uptake at the cellular level, induced apoptosis of tumor cells, and increased in vivo bioavailability of ICG at the tumor site [115]. The scheme and the mechanisms involved in utilizing the pH sensitive polymer, HA mediated inorganic enzyme and ICG loading to produce ROS are shown in Figure 14.

Another work depicted utilization of SPIONPs (Fe_3_O_4_) for targeted delivery incorporated with cerium oxide (CeO_2_) on the surface of NPs to generate ROS in the tumor environment, inducing oxidative stress and selective killing of cancer cells. Additionally, HA was used to coat the CeO_2_ surface and target CD44-overexpressing tumor cells, while ^nat^Zr was chelated on the Fe_3_O_4_-CeO_2_ surface to enable labeling with the radioisotope ^89^Zr. Furthermore, good dispersibility of the HA coated NPs showed that CeO_2_ generated ROS and targeted delivery [116].

**Figure 15:**
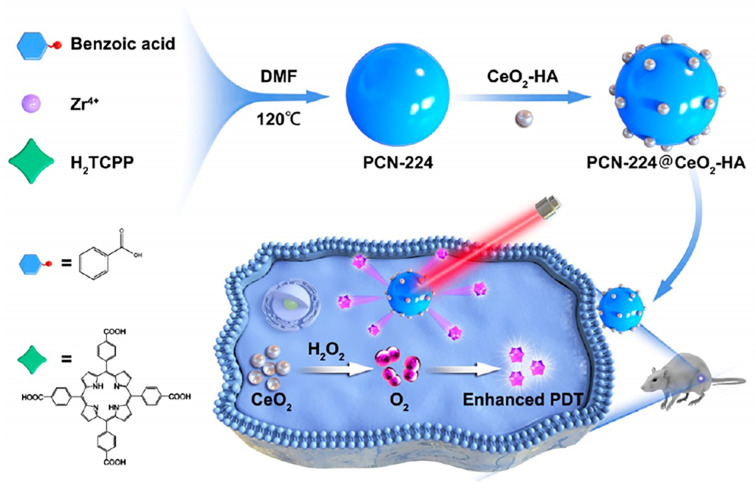
Schematic representation to obtain HA-CeO_2_-MOF and PDT treatment. Reproduced from [117].

Although photodynamic therapy (PDT) has shown better results in cancer treatment, its effectiveness has been limited by hypoxic tumors, poor targeting, and photosensitizer (PS) aggregation. To address all these drawbacks, HA-modified CeO_2_ decorated with metal-organic frameworks (MOFs) to produce HA-CeO_2_-MOF and the resulting PDT treatment are shown in Figure 15. The CeO_2_ catalyzes H_2_O_2_ to produce O_2_, resolving hypoxia issues, and HA targets the CD44 receptor expressed on tumor cell membranes. When incubated with HA-CeO_2_-MOF under laser irradiation, the growth of tumor cells 4T1 and MCF-7 was distinctly controlled, while the survival of normal cell LO2 was nearly unchanged. Importantly, HA-CeO_2_-MOF was effectively aggregated within the tumor area 12 h after injection and remarkably inhibited tumor growth under laser irradiation [117].

Ulcerative colitis (UC) is a challenging chronic nonspecific inflammatory bowel disease characterized by rapid progression. The high expression of myeloperoxidase (MPO) in colonic ulcers of UC patients results in an abundance of macrophages and ROS. In this respect, Gao et al. developed an electrostatically assembled MPO targeting HA/serotonin/(5-HT) ceria nanoenzyme (HA-5-HT-CeO_2_) to address existing challenges. By using CeO_2_ NPs, 5-HT and HA mediated to achieve dual targeting effects of MPO and the macrophage CD44+ receptor, the HA-5-HT-CeO_2_ was able to locate the inflammatory site and eliminate O_2_, H_2_O_2_, and ROS. This strategy successfully repaired the intestinal epithelial barrier by specifically targeting inflammatory factors. In vitro pharmacodynamic investigations and animal models of acute colitis indicated that HA-5-HT-CeO_2_ demonstrated superior efficacy in reducing inflammation and treating ulcerative colitis compared to conventional drugs [118]. Overall, the core materials used, other substituents and key outcomes are summarized in Table 5.

## 8. Potential Challenges Involved in Clinical Translations

The advancement of M-NPs is rapidly evolving, providing alternative approaches to cancer treatment and enhancing the effectiveness of various cancer therapies. Numerous in vitro and in vivo investigations have reported encouraging outcomes in the treatment of various types of cancer by utilizing HA-modified M-NPs and other substituents with inherent anticancer properties, or metallic nanoplatforms in combination with other therapeutic modalities. The growing body of literature demonstrates the potential of these approaches in cancer treatment. The application of controlled release and targeted systems that can be triggered by factors such as pH, temperature, electromagnetic waves, light, and enzymes provides vital precision in the delivery of chemotherapeutic agents, resulting in enhanced accumulation of drugs in tumor tissues and improved therapeutic efficacies [26]. Additionally, detailed and comprehensive investigation is required for the transition of noble and non-noble M-NPs from the laboratory to the clinic. While the earliest gold M-NPs are gradually being utilized in clinical trials after undergoing in vivo/in vitro studies, silver, palladium, and platinum are still in their infancy stages, with more structural and functional possibilities yet to be explored [119]. On the other hand, HA is widely utilized in active tumor targeting and hyaluronidase degradation. The interaction between HA and CD44 is greatly influenced by the molecular weight of HA, and the protein corona may impede degradation through hyaluronidase, or hinder the interaction between specific ligand and receptors. A sdual stimuli responsive strategy, such as response to pH, GSH, and NIR, may be a solution. Additionally, the cross influence of hyaluronidase degradation and CD44 binding with HA in the tumor should be evaluated. However, the targeting capacity of HA may not be significantly impacted by hyaluronidase degradation, as CD44-mediated internalization occurs quickly when the HA or hybrids bind with tumor cells [22]. These are some of the concerns that need to be addressed. Finally, there are some clinical trials utilizing HA in cancer therapy, wherein HA-irinotecan entered Phase II trials in treating metastasis colorectal cancer. Similarly, another Phase II trial involved HA-irinotecan with carboplatin for intravenous injection. However, further clinical trials are required for the inclusive evaluation of HA-modified M-NPs and other substituents for cancer therapy in the near future [25]. The transition from clinical to industrial application of HA-modified M-NPs in cancer treatment poses several challenges due to the complexity of the process. Thus, we outline some of the key factors that contribute to the complexity of this transition and the considerations involved.

1. Scale-up process: Moving from laboratory-scale synthesis to large-scale production of HA-modified M-NPs requires optimization of manufacturing processes. Factors such as reproducibility, batch-to-batch consistency, and quality control need to be addressed. Scaling up the production process, while upholding the desired physicochemical properties and functionalization, is crucial for industrial applications.
2. Cost: Industrial production regularly entails cost-effective strategies. The selection of raw materials, purification techniques, and synthesis methods should be optimized to minimize costs without compromising quality and performance. Further, economical scale-up processes need to be developed to ensure affordability for widespread cancer treatment.
3. Stability and shelf-life: Ensuring the stability and extended shelf-life of HA-modified M-NPs is crucial for industrial applications. Stability studies should be conducted to evaluate the NPs’ physicochemical properties, such as size, surface charge, and drug-loading capacity, over time. The development of appropriate storage and transportation conditions is essential to preserve the therapeutic efficacy.
4. Regulatory considerations: Regulatory guidelines and requirements play a vital role in the transition from clinical to industrial use. Comprehensive preclinical and clinical studies should be conducted to assess the safety, efficacy, and toxicity profiles of HA-modified M-NPs. Data on pharmacokinetics, biodistribution, and long-term effects are essential for regulatory approval. Furthermore, compliance with good manufacturing practices (GMPs) and other relevant protocols is necessary for industrial-scale productions.
5. Quality Control and characterization: Industrial production requires stringent quality control measures to ensure consistency and reproducibility. Robust analytical methods should be established for accurate characterization, including size distribution, surface chemistry, and drug-loading efficiency. Furthermore, standardization of characterization techniques is essential for batch-to-batch consistency and comparability.
6. Scalability of functionalization: HA-modified M-NPs can be functionalized with various ligands, targeting moieties, or therapeutic agents to enhance their specificity and efficacy. Thus, developing scalable methods for functionalization and achieving uniform surface handling are critical challenges. The biocompatibility of different functionalization strategies with large-scale production needs to be evaluated.
7. Manufacturing partnerships and collaboration: Establishing collaborations between research institutions, pharmaceutical companies, and manufacturing facilities is crucial for the successful transition to industrial application. Collaboration can help leverage expertise, resources, and infrastructure required for large-scale production, quality control, and regulatory compliances.

Overall, the clinical to the industrial transition of HA-modified M-NPs in cancer treatment is a complex process that involves optimization of manufacturing processes, cost considerations, stability, regulatory compliance, quality control, and collaborations. Addressing these challenges will facilitate the translation of this promising technology into practical and accessible solutions for cancer patients.

## 9. Conclusions and Future Prospects

In conclusion, we summarized past and most recent progress in the application of HA-modified M-NPs and other substituents in various cancer therapy applications utilizing different cancer therapeutic approaches. The abundance of literatures in recent years makes it evident that using HA-modified M-NPs and other substituents as biomaterials to target different tumors is a promising and attractive approach for enhancing cancer therapy. Definitely, there is a dearth of research on the biodistribution, toxicity, and availability of HA-modified M-NPs and other substituents under physiological conditions. A more in-depth study of these parameters is imperative for clinical implementation. Overall, the development of cancer therapeutic systems is a multidisciplinary field that necessitates expertise from a range of fields, such as chemistry, material science and engineering, nanotechnology, and medicine. Therefore, several experts from diverse domains are collaborating to design innovative cancer therapeutic systems that are clinically efficient and have minimal side effects. Such systems are expected to enhance human health in the near future. Some of the future directions listed are below:

1. Targeted drug delivery: HA modification of M-NPs and with other substituents enables targeted drug delivery to cancer cells. Future research can focus on developing multifunctional NPs that encapsulate other therapeutic agents, such as chemotherapeutic drugs and small interfering RNA (siRNA). The incorporation of targeting ligands or antibodies on the surface could enhance specificity towards cancer cells, minimizing off-target effects.
2. Imaging and diagnosis: HA-modified M-NPs can serve as excellent imaging agents for cancer diagnosis. The unique optical, magnetic, and photoacoustic properties of M-NPs can be exploited to develop imaging probes for early cancer detection, precise tumor localization, and monitoring of therapeutic responses. Future research should explore the integration of imaging modalities with therapeutics, allowing simultaneous diagnosis and treatment.
3. Photothermal therapy: M-NPs possess photothermal properties, converting light into heat, which can be utilized for targeted cancer therapy. HA modification enhances tumor accumulation and internalization of M-NPs, making them an ideal platform for photothermal therapy. Future studies can focus on optimizing NP design, selecting appropriate light sources, and investigating the synergistic effects of combining photothermal therapy with other treatment modalities.
4. Immunotherapy developments: HA modification of M-NPs holds potential in modulating the tumor microenvironment and enhancing immunotherapy approaches. The immune response can be stimulated by incorporating immune modulators, such as cytokines or immunomodulatory agents, onto the surface of NPs. Moreover, M-NPs can act as adjuvants to promote antigen presentation and improve the efficacy of cancer vaccines. Future research can explore these strategies and investigate the immunomodulatory mechanisms to develop personalized cancer immunotherapies.
5. Theranostic platforms: Integration of diagnosis and therapy into a single platform, known as theranostics, is a promising approach in cancer treatment. HA-modified M-NPs can serve as versatile theranostic agents by combining imaging capabilities, targeted drug delivery, and therapeutic modalities. Future research should focus on developing more multifunctional nanoplatforms that can be precisely controlled and optimized for personalized cancer therapy.
6. Safety and toxicity considerations: As with any novel therapeutic approach, the safety and toxicity profiles of HA-modified M-NPs and other substituents must be thoroughly evaluated. Future research should investigate the long-term effects, biodistribution, and potential adverse reactions associated with the use of these systems in cancer therapy.

## Figures and Tables

**Table 1:** Summary of the importance of Au-HA as core material along with other substituents and key outcomes for the treatment of various cancers using different therapeutic approaches.

Sr.no.	Core Materials	Other Substituents	Key Outcomes	Ref.
	HA-modified Au			
1.		LAP-PLA-PEG-PEI	High loading efficiency of DOX at 91.0 ± 1.8% and pH-sensitive sustained release Effectively suppresses tumor growth with reduced side effects	[62]
2.		mSiO_2_-RGD	High loading capacity of DOX around 20.16% Enhanced cellular uptake and targeted to ovarian cancer cells through dual mechanisms	[63]
3.		PEG-Aptamer	DOX loading was 270 molecules per Au-NPs Designed hybrid system was ten times more potent than DOX alone	[64]
4.		FA	DOX loading capacity of around 7.1 wt.% observed Induced cell apoptosis under NIR irradiation and in vivo experiments resulted in the complete elimination of tumors without causing severe side effects to normal tissues	[65]
5.		-	SN38 loading capacity of 17.4% observed and in vitro release studies showed that drug release at acidic conditions was faster when compared with the physiological pH environments	[66]
6.		HSN	Under NIR, excellent endocytosis in cancer cells without inducing cytotoxicity was observed	[67]
7.		-	Designed systems were more cytotoxic than free cisplatin In vivo experiments showed significant antitumor efficacy when combined with near-infrared laser treatment	[68]
8.		PGMA	Displayed superior selective targeting toward cancer cells Excellent photothermal outcomes and improved efficacies against cancer cells when compared to normal cells	[69]
9.		PEI-PEG	Proved to have good biocompatibility, highlighting significant role as a new strategy for cancer treatment to overcome MDR	[70]
10.		CBSA	Successful in reducing tumor growth by 95.3% and inhibiting the growth of lung metastasis by 88.4%	[71]
11.		-	Delivered to tumor tissues with an accumulation ratio of 12.8%. Completely eliminated tumors without obvious side effects	[72]
12.		-	The formulation and the bioactivity of the released RA was demonstrated in a reporter cell line expressing luciferase controlled by the RA receptor	[73]
13.		DHHC	Evident pH-responsive drug release and efficient internalization by MCF-7 cells and proved synergistic therapeutic effects	[74]
14.		PAMAM	Remarkable intratumoral penetration and synergistic radio-chemotherapeutic effects	[75]
15.		DAHA-HECS	Proved to have 4.1% of DOX content and exhibited pH/NIR drug release behaviors. Effectively taken up by MCF-7 breast cancer cells and displayed superior efficacy in eliminating cancer cells	[76]
16.		pSiO_2_	The Amox loading content was 18.2% and release rate regulated by redox and enzymatic degradation The pSiO2-Au/HA exhibited remarkable photothermal conversion efficiency	[77]
17.		LA	Around 5.0% of DOX loading capacity observed and enhanced cancer cell-killing and tumor growth inhibiting abilities	[78]
18.		HCA	Highly selective and showed remarkable phototoxicity	[79]
19.		MSS-TPGS	Functionalization improved hemocompatibility and selectivity towards cancer cells Induced the death of HeLa cancer cells through an on-demand photothermal effect	[80]
20.		FA	Proved to have good biocompatibility, stability, and loading capacity for Ce6, DOX were 11.3 and 10.00% The combination of chemotherapy and PDT in the nanocomposite resulted in significant cancer cell death upon exposure to laser irradiation	[81]
21.		RA-dPG	The expression of stemness genes and CSC tumorsphere development are notably reduced In vivo study eliminated tumor growth and CSCs, indicating higher anticancer activity and effective CSCs suppression.	[82]
22.		-	Easily accumulated in CD44-overexpressing MDR breast cancer cells via CD44-HA recognition The CAT catalyzed the decomposition of endogenous H2O2 to generate O2, thus mitigating hypoxia and ensuring high PDT efficiency	[83]
23.		PDA	DOX loading content was around 10.00 wt.% and display excellent photothermal performance under NIR irradiation Destroyed tumor cells and accelerated the release of DOX	[84]

**Table 2:** Summary of the importance of Ag-HA as core materials along with other substituents and key outcomes for the treatment of various cancers using different therapeutic approaches.

Sr.no.	Core Materials	Other Substituents	Key Outcomes	Ref.
1.	HA modidied Ag	-	Significantly inhibited leukemia cell viability by inducing apoptosis via specific binding of HA with CD44 receptors that were overexpressed on the cell surface Reduced systemic toxicity. Improvement of leukemia-specific chemotherapy	[85]
2.		Fe	Proved to have synergistic anticancer properties Improved the transportation of the active components within HeLa spheroids	[86]
3.		SiO_2_-EGDMA	Induced cancer cell apoptosis	[87]

**Table 3:** Summary of the importance of Pt/Pd-HA as core materials along with other substituents and key outcomes for the treatment of various cancers using different therapeutic approaches.

Sr.no.	Core Materials	Other Substituents	Key Outcomes	Ref.
	HA-modified Pt			
1.		-	In vitro experiment validated CD44-overexpressing cancer cells were internalized more effectively In-vivo studies proved superior efficacy in inhibiting tumor growth	[88]
2.		DMSN-CDs	ROS generation and other radicals under hypoxic conditions	[89]
3.		Pd	ROS generation and induced cell apoptosis In vivo experiments demonstrated excellent treatment efficacy on 4T1 and h22-tumor-bearing mouse models	[90]
	HA-modified Pd			
1.		Se	In vitro studies proved good photothermal effect, -OH scavenging ability, effectively inhibited macrophage infiltration, ROS production, and cytokine-mediated inflammation Completely stopped the inflammatory response in the joints of mice with an induced RA model and stopped joint degradation	[91]
2.		BTO-MnO_2_	Multistep improved SDT process, activated by TME decomposition Enhanced SDT-CDT therapies	[92]

**Table 4:** Summary of the importance of Fe-HA as core material along with other substituents and key outcomes for the treatment of various cancers using different therapeutic approaches.

Sr.no.	Core Materials	Other Substituents	Key Outcomes	Ref.
	HA-modified Fe			
1.		CCh	Biological studies showed that hybrid systems could be easily internalized into cells and did not exhibit cytotoxicity at the tested concentration	[93]
2.		-	Improved cellular uptake and accumulation in CD44 HA receptor-overexpressing MDA-MB-231 cells	[94]
3.		PEI-BSA-QD	In vitro studies revealed specific cellular uptake The tumor was successfully ablated in nude mice after intravenous injection in the presence of NIR	[95]
4.		-	Notable cellular uptake and accumulation. In vivo antitumor efficacy demonstrated in mice confirming substantial therapeutic potentials	[96]
5.		-	Around 75% of HCPT drug encapsulation witnessed. In vitro and in vivo experiments demonstrated remarkable magnetic tumor targeting and effective tumor cell ablation Notably, no systemic toxicity was observed	[97]
6.		-	Proved to have DTX drug with loading efficiency of 10.9%. Enhanced cellular uptake and endocytosis Superior localized photothermal ablation	[98]
7.		PDA	In vitro and in vivo showed preferential tumor accumulation, improved specificity to target tumor cells, pH- and laser-responsive releases, and a high tumor cell-killing efficiency	[99]
8.		PEG	The MTX loading efficiency was around 87.7% and was stable in physiological conditions for up to 8 days Proved to significantly induce apoptosis in MDA-MB-231 cell lines	[100]
9.		Pt, APTES	An LND loading efficiency of 6.3% was obtained In vivo results confirmed enhanced uptake of SPANs in the brain after intranasal administration with improved nasal and mucus penetration	[101]
10.		PEI-mPEG	The cellular uptake analysis showed that MIAPaCa-2 cells, which overexpress the CD44 receptor, were specifically internalized	[102]
11.		PC-NH_2_ (porous carbon)	Exhibited exceptional biocompatibility and efficient photothermal transformation capability Showed DOX drug loading capacity of around 27% In vivo T2-weighted MR imaging confirmed the accumulation in the tumor	[103]
12.		LDH	Hybrids demonstrated a 10-fold increase in r1 relaxivity compared to Fe3O4 NPs Proved to have 57.65% DOX loading capacity Validated by pH-responsive release behavior, which showed targeted tumor inhibition effects, tumor penetration and significantly enhanced MR imaging ability	[104]
13.		APTES-Phosphatidylcholine (PC)	Proved to have good antitumor efficacy for MDR cancer therapy with minimal cardiotoxicity Delivered DOX to the xenograft tumor model	[59]
14.		-	The ILP value of Fe3O4-HA NPs was about 3.5 nHm2/kg, which was about 25-fold more when compared with commercially available Fe3O4 NPs	[105]
15.		PLGA-OA	By varying the amount of cisplatin, it was possible to obtain loading capacity differences from 5.3 to 18.00% Superior intracellular uptake efficiency and active targeting of U87 cancer cells In a xenograft tumor model in nude mice, treatment with via tail vein injection, resulted in the lowest tumor growth rate	[106]
16.		Peptide	DOX loading capacity was around 21.7% Proved to have good biostability and a preferred drug release profile at low pH After intravenous injection in MC38 tumor-bearing mice model, exhibited 2.5 times higher tumor-targeting effect	[107]
17.		β-cyclodextrin	A pH-sensitive release performance was observed	[108]

**Table 5:** Summary of the importance of Zn-HA and Ce-HA as core materials along with other substituents and key outcomes for the treatment of various cancers using different therapeutic approaches.

Sr.no.	Core Materials	Other Substituents	Key Outcomes	Ref.
	HA-modified Zn			
1.		PEG-QDs	Around 52% of DOX loading content observed Induced hyperthermia and promoted the release of DOX when exposed to NIR irradiation	[109]
2.		-	Validated with prolonged circulation time in the bloodstream, enhanced accumulation in the tumor site, and remarkable antitumor efficacy	[110]
3.		-	Exhibited anti-cancer effects on three different cancer cells and intracellular ROS were observed	[111]
4.		Mn-Zn-PEG-PCL	In the A549 subcutaneous tumor xenografts model, MRI proved enhancement in the tumor The IHC confirmed enhanced tumor oxygenation	[112]
5.		PLGA	Destroyed the tumors of nude mice upon laser irradiation	[113]
	HA-modified Ce			
1.		PEI	Proved to have superior anticancer effects and responsible for the generation of ROS	[114]
2.		PEI	Effectively improved the hypoxic microenvironment of the solid tumor Induced apoptosis of tumor cells, and increased in vivo bioavailability at the tumor site	[115]
3.			Generated ROS in the tumor environment, inducing oxidative stress and selective killing of cancer cells	[116]
4.		MOF	Remarkably inhibited tumor growth under laser irradiation	[117]
5.			Demonstrated superior efficacy in reducing inflammation and treating ulcerative colitis compared to conventional drugs	[118]

## Data Availability

Not applicable.
